# Interleukin-10 suppresses lipid metabolism-mediated intestinal inflammation

**DOI:** 10.1093/lifemeta/loae011

**Published:** 2024-03-26

**Authors:** Tristram A. J. Ryan, Ivan Zanoni

**Affiliations:** Division of Immunology and Division of Gastroenterology, Harvard Medical School and Boston Children’s Hospital, Boston, MA 02115, United States

## Abstract

In a recent paper published in *Nature*, York *et al*. reported that the anti-inflammatory cytokine IL-10 regulates sphingolipid metabolism to limit NF-κB-mediated inflammation. Deletion of *Il10* in mice, or genetic mutation of *IL10* in humans, predisposes to inflammatory bowel disease, which may be overcome by restoring homeostatic sphingolipid metabolism

The host response to infection is orchestrated by broad-ranging defense systems including rapid inflammation to target and neutralize invading pathogens, followed by a resolution phase to limit host damage. Key components of the inflammatory milieu include cytokines, which are heterogenous signaling proteins that trigger or dampen activation of immune pathways — a primary host defense mechanism — to combat the offending pathogen. A rapid return to homeostasis upon pathogen clearance is critical to avoid a pathological overamplification of this host-derived inflammatory response, which may drive inflammatory diseases, such as inflammatory bowel disease (IBD).

One of the best-studied anti-inflammatory cytokine families is the interleukin-10 (IL-10) superfamily — a group of highly pleiotropic cytokines and their associated receptors — which can act via activation of Janus kinase-signal transducer and activator of transcription (JAK-STAT) signaling, a central component in host defense during infection or injury (1). The IL-10 superfamily of immune mediators includes the IL-20 subfamily (comprising IL-19, IL-22, and other cytokines) and type III interferons (IFNλ1–4 in humans), which have been implicated in regulating gut homeostasis (2,3). IL-10 itself is critical for suppressing excessive activation of the immune response during infection, limiting host damage. A prime example of this is the severe and early-onset IBD that develops in mice (4) and humans (5) lacking functional IL-10 or *IL10RA*/*IL10RB* (encoding the IL-10 receptors) genes, highlighting the protective role that IL-10 plays in maintaining intestinal homeostasis.

The precise mechanism by which IL-10 exerts its anti-inflammatory functions is hitherto unknown. Nevertheless, IL-10 signaling in macrophages — an innate immune cell type that is important in attacking invading pathogens and mounting the host response to infection — has been shown to protect against colitis development (6,7). In a recent study published in *Nature*, York *et al*. (8) reported that IL-10 reduces intestinal inflammation in mice by tightly regulating lipid metabolism in macrophages (Fig. 1). A well-characterized feature of inflammatory macrophages is the crosstalk between macrophage metabolism and innate immune pathways, a process termed immunometabolism, and the authors found that downstream of Toll-like receptor (TLR) 2 (a pattern recognition receptor for gram-positive bacteria including *Staphylococcus aureus*) activation, *Il10*-knockout (KO) mouse macrophages and mice exhibit alterations in their lipid compositions compared with wild-type (WT) counterparts. *Il10* deficiency resulted in altered expression of components of sphingolipid biosynthesis — which is critical for cell membrane formation — via upregulation of the *de novo* ceramide synthesis pathway leading to accumulation of ceramides and a decrease in sphingomyelins.

York *et al*. next assessed whether ceramide accumulation might explain the proinflammatory genotype which is characteristic of *Il10*-KO macrophages. They found that exogenous saturated very long chain (VLC) ceramides, but not long chain ceramides, recapitulated the elevated inflammatory gene expression in WT macrophages, but only when administered after TLR2 activation, indicating the importance of *Il10* induction and signaling in dampening lipid metabolism-mediated inflammation. To further probe the role of VLC ceramides in inflammation, the authors generated mice lacking ceramide synthase 2 (CerS2), a protein critical for VLC ceramide synthesis. Macrophages lacking VLC ceramide synthesis exhibited reduced proinflammatory gene expression in response to TLR2 activation, providing evidence for a genetic contribution of VLC ceramides to inflammation. Importantly, macrophages lacking both IL-10 and VLC ceramide synthesis expressed reduced inflammatory gene expression *in vitro*, but this response was impaired by the addition of exogenous VLC ceramides. Furthermore, mice lacking both IL-10 signaling and VLC ceramide synthesis showed reduced immune cell infiltration into the colon compared with *Il10*-KO mice, highlighting the proinflammatory effects of VLC ceramide accumulation in the context of impaired IL-10 signaling in colitis. The authors attributed this in part to the reduced inflammatory macrophage population and therefore more tolerogenic macrophages, which they identified as a key suppressor of colonic inflammation in the double-KO mice.

The authors next delved deeper into the regulation of sphingolipid metabolism by IL-10 and found decreased expression of a gene involved in monounsaturated fatty acid (MUFA) generation in the absence of IL-10. MUFAs, such as oleic acid, are described to have beneficial, anti-inflammatory properties and are notable for being enriched in the Mediterranean diet which is associated with decreased risk of obesity, cardiovascular disease, and Alzheimer’s disease. The authors identified decreased oleic acid synthesis and total content in *Il10*-KO macrophages following TLR2 activation. Then they elegantly ameliorated the proinflammatory genotype in *Il10*-KO macrophages by exogenous addition of MUFAs, but not with polyunsaturated fats. MUFAs were also found to suppress saturated VLC ceramide abundance, demonstrating that IL-10 controls macrophage lipid metabolism and restrains their inflammatory activity via MUFA synthesis.

Consistent with this protective role attributed to MUFAs in the context of TLR2 activation, mice lacking MUFA synthesis displayed increased intestinal inflammation which was exacerbated in a model of dextran sodium sulfate-induced colitis, a commonly employed mouse model that mimics many aspects of human IBD. Increased numbers of macrophages were identified in the colons of mice lacking MUFA synthesis, which the authors linked to increased activation of adaptive immune cells called T cells, which are important for killing pathogens and can act as a memory for the immune system upon re-encounter of a previously recognized pathogen. This crosstalk between macrophages and T cells triggered elevated proinflammatory cytokine production in the colons of mice that could not produce MUFAs, which likely contributes to the intestinal pathology that occurs with dysregulated sphingolipid metabolism. Collectively, this indicates that IL-10 mediates MUFA synthesis in macrophages to orchestrate a potent anti-inflammatory response across multiple immune cell types to limit intestinal inflammation.

York and colleagues then established that saturated VLC ceramides triggered a prolonged activation of a major transcription factor in macrophages, nuclear factor kappa-light-chain-enhancer of activated B cells (NF-κB), which signals to induce proinflammatory cytokine production, enhancing the immune response and inflammation. Macrophages lacking REL, a major component of the NF-κB family, were protected against VLC ceramide-mediated inflammation. Notably, the absence of REL did not alter ceramide production or uptake in macrophages, suggesting NF-κB activation therefore occurs as a consequence of – but not a mediator for – VLC ceramide synthesis. Macrophages lacking MUFA synthesis also exhibited increased NF-κB activation and this process was limited by the addition of exogenous MUFAs. Linking the components of this pathway together, the authors finally found that mice lacking both IL-10 and REL had reduced colonic inflammation and immune cell activation when compared with *Il10*-KO mice, suggesting that NF-κB activation is a key trigger for the dysregulated sphingolipid metabolism-mediated colitis that occurs in the absence of IL-10 signaling (Fig. 1).

This study elucidates a previously unacknowledged route by which IL-10 exerts its anti-inflammatory effects via regulation of sphingolipid metabolism, particularly in the context of IBD, which may be exploited for future treatment approaches. Subsequent studies should assess lipid metabolism in IBD patients, particularly in those individuals with dysfunctional IL-10 signaling, to assess VLC ceramide and MUFA synthesis. Altered lipid metabolic profiles – which have been shown to modify the immune response (9) – in these patients may support further exploration of MUFAs, and perhaps the Mediterranean diet – which has previously been shown to be beneficial for alleviating inflammation in colitis (10) – for the management of idiopathic conditions such as IBD. Elucidating the exact upstream mechanism by which IL-10 restrains VLC ceramide accumulation may also aid in refining recombinant IL-10 therapies which have been tested in the clinic for colitis but ultimately failed to meet their endpoints due to lack of precision and efficacy.

Dysregulation of the IL-10-sphingolipid metabolism axis may also contribute to pathology in other clinical conditions such as COVID-19, where aberrant IL-10 production as well as alterations in sphingolipid metabolism and signaling have been reported. Altogether, the study by York and colleagues establishing control of lipid metabolism by IL-10 may have broad consequences for the study and therapy of multiple inflammatory conditions.

## Figures and Tables

**Figure 1:**
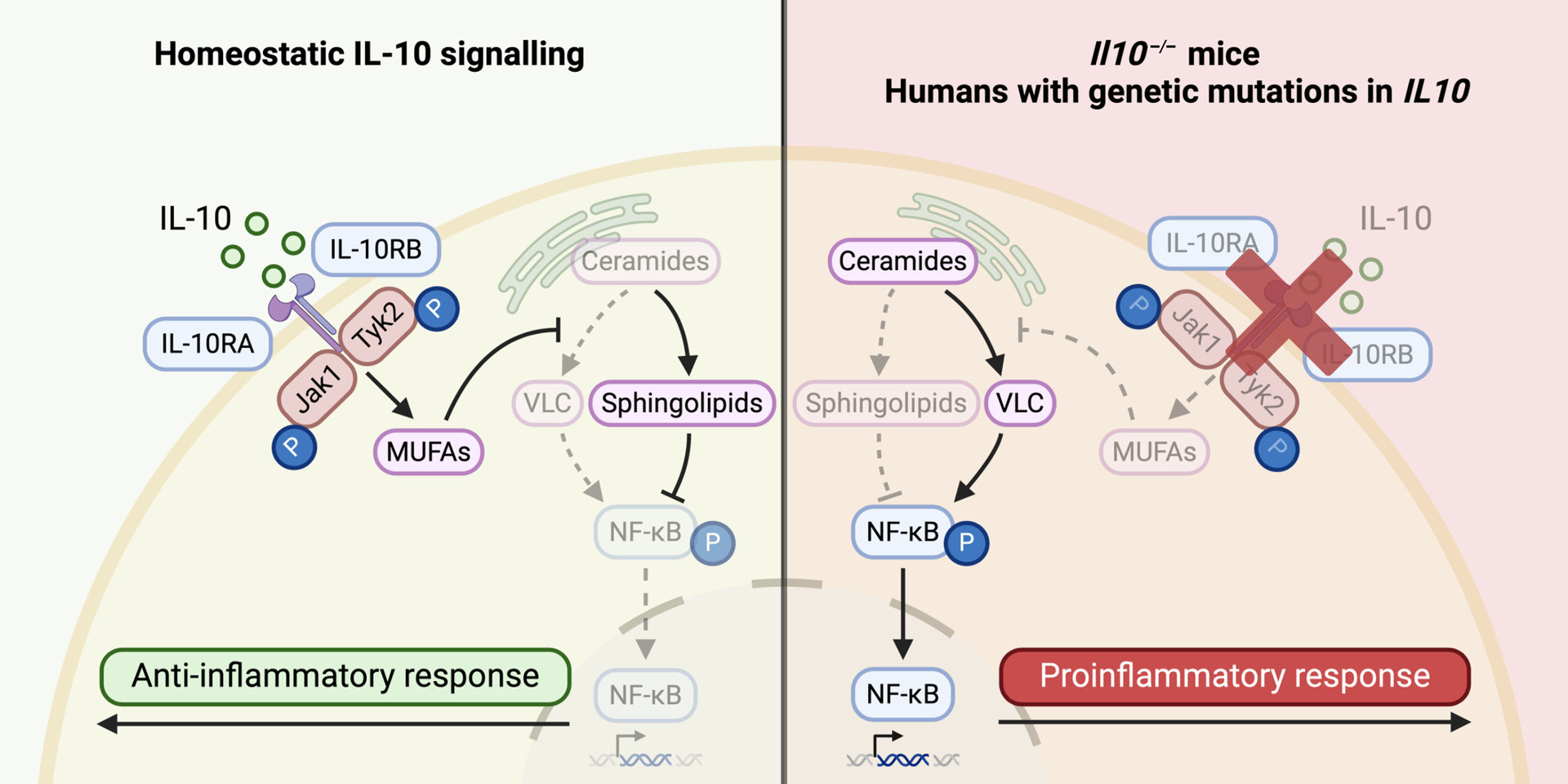
IL-10 drives the synthesis of MUFAs in macrophages to limit the production of proinflammatory VLC ceramides to regulate sphingolipid metabolism, maintaining homeostasis. However, in mice or humans with IL-10 signaling deficiency, IL-10-mediated MUFA production is abrogated, facilitating the accumulation of proinflammatory VLC ceramides which disrupts sphingolipid metabolism. This can result in prolonged NF-κB activation, driving the detrimental and proinflammatory phenotype associated with inflammatory bowel disease.
